# The Value of Mandibular Indices on Cone Beam Computed Tomography in Secondary Causes of Low Bone Mass

**DOI:** 10.3390/jcm13164854

**Published:** 2024-08-16

**Authors:** Ioana Ruxandra Poiană, Ramona Dobre, Silviu-Mirel Pițuru, Alexandru Bucur

**Affiliations:** 1 Faculty of Dentistry, “Carol Davila” University of Medicine and Pharmacy, 050474 Bucharest, Romania; silviu.pituru@umfcd.ro (S.-M.P.); alexandru.bucur@umfcd.ro (A.B.); 2 Faculty of Medicine, “Carol Davila” University of Medicine and Pharmacy, 050474 Bucharest, Romania; ramona.dobre@umfcd.ro; 3 Department of Endocrinology, National Institute of Endocrinology C. I. Parhon, 011853 Bucharest, Romania

**Keywords:** secondary and primary osteoporosis, menopause, cone beam computed tomography, mandibular indices

## Abstract

**Background:** As implant treatment cases increase, many cases of failure/side effects also occur. Generally, dental clinics determine the density of the jawbone using cone beam CT (CBCT). Considering the known potential role of this tool for bone assessment in primary osteoporosis, this study evaluated patients with secondary endocrine causes of low bone mass. **Methods:** The study included 83 patients with endocrine causes of osteoporosis who were evaluated by dual-energy X-ray absorptiometry (DXA), trabecular bone score (TBS), and mental foramen (MF) region CBCT. The following CBCT indices were measured: anterior (A)—thickness of inferior mandibular cortex 10 mm anterior from MF; molar (M)—thickness of inferior mandibular cortex 10 mm posterior from MF; posterior (P)—thickness of inferior mandibular cortex 25 mm posterior from MF; symphysis (S)—thickness of inferior mandibular cortex equidistant from the centers of right and left MF. **Results:** The highest correlation coefficient in the secondary causes group was between the A index and the lumbar BMD (r = 0.375, *p* = 0.001) and the P index and the femoral neck BMD (r = 0.38, *p* = 0.001). Hypercortisolism seems to be the most predictable cause of secondary osteoporosis using the A, M, and P indices. The A, M, and P indices showed predictive values of the bone micro-architecture that was evaluated using TBS score, and were statistically significant. The symphysis index does not significantly predict osteoporosis or impaired bone micro-architecture. **Conclusions:** These findings support the potential usefulness of A, M, and P CBCT-derived radiomorphometric mandibular indices in secondary osteoporosis, underlining the well-known effects of these pathologies on bone micro-architecture rather than bone quantity.

## 1. Introduction

Osteoporosis is a systemic skeletal disorder characterized by decreased bone mass and the deterioration of bone tissue, which is a significant clinical challenge for practitioners. This condition can arise from primary causes, such as age-related bone loss and postmenopausal estrogen deficiency, or secondary causes (see Table 1), particularly endocrine disorders like hyperparathyroidism, hyperthyroidism, and Cushing’s syndrome [1,2]. Differentiating between primary and secondary causes is crucial for effective management, as the underlying pathophysiology and treatment approaches vary significantly. Secondary endocrine causes of osteoporosis not only accelerate bone resorption but also impair bone micro-architecture, contributing to an increased risk of fractures compared to postmenopausal osteoporosis [3].

Dental radiography is essential in clinical dentistry, with cone beam computed tomography (CBCT) [4,5] being an advancing diagnostic tool and allowing more accurate and informed diagnosis and treatment planning. CBCT’s advantage of multiplanar reconstruction allows visualization without the superimposition of structures [6,7], facilitating a more precise evaluation of bone architecture and dimensions before dental implants [8,9], although multisource cone beam computed tomography CBCT (ms-CBCT) has been shown to overcome some of the inherent limitations of conventional CBCT [10]. Quantitative CBCT-based BMD measurement is a valuable tool for assessing bone quality and density in the diagnosis and treatment of various structures, offering high-quality BMD images that support accurate and effective treatment [11].

Unlike conventional radiography, CBCT provides three-dimensional imaging with high resolution and a relatively low radiation dose and can offer detailed insights into bone architecture. This particularly can be useful in the context of osteoporosis, where the precise assessment of bone density and structural integrity is critical [4]. In a study that compared bone micro-architecture parameters of bone samples scanned using micro-CT (µCT) to those obtained by using CBCT, 8 out of 16 evaluated bone parameters showed a significant correlation. However, it was seen that there was considerable uncertainty regarding the stability of these parameters [12].

Radiomorphometric indices, derived from imaging studies, can be useful in assessing low bone mass and further predicting the fracture risk. These indices, such as the mandibular cortical width and panoramic mandibular indices, can be evaluated using CBCT, enhancing the ability to detect osteoporosis [13] by quantifying bone micro-architecture and density and providing valuable metrics for clinicians to monitor disease progression and treatment efficacy. Our purpose was to evaluate the correlations between radiomorphometric mandibular indices and bone mass density in patients with secondary causes of low bone mass, regarding both the quantity and the micro-architecture of the bone assessed by dual-energy X-ray absorptiometry (DXA) and the trabecular bone score (TBS). The CBCT-derived indices evaluated are very similar to the MCW used in panoramic radiographs but in different locations in the mandible [14,15].

This is among the first studies to evaluate the potential use of CBCT-derived radiomorphometric indices in patients with secondary endocrine pathologies known to cause low bone mass.

## 2. Materials and Methods

The present study included 83 patients with secondary endocrine pathologies with a known effect on bone mass: hyperthyroidism (*n* = 10), hyperparathyroidism (*n* = 4), hypercortisolism (*n* = 14), acromegaly (*n* = 12), secondary or type 2 diabetes mellitus (*n* = 40), and on-going treatment with aromatase inhibitors for breast cancer (*n* = 15), with normal BMD, osteopenia or osteoporosis, with or without specific anti-osteoporotic treatment. The inclusion criteria were known endocrine pathology with bone effects, DXA with TBS evaluation, biochemical evaluation, and CBCT evaluation. There were 73 female patients and 10 male patients. The exclusion criterion was the presence of systemic diseases affecting bone metabolism (neoplasia, osteomalacia, history of rickets, severe renal failure, liver failure, malabsorption disorders; usage of medications interfering with bone density (except for glucocorticoids and aromatase inhibitors)).

The patients included in the study were future or previous candidates for a dental implant and they were required to do a CBCT as part of the preimplant protocol commonly used in our country (most of the institutions are private practices with personalized protocols, but CBCT evaluation is commonly used in most of the dental clinics). The patients were evaluated in close collaboration with an important private provider of dental Imaging with expertise in CBCT imaging and, respectively, the National Institute of Endocrinology, a public hospital, that facilitates hundreds of patients with metabolic bone pathologies every year.

Written informed consent was obtained from the patients before the study. The study was approved by the Ethics Committee of “C. I. Parhon” National Institute of Endocrinology, Bucharest, Romania (no. 4/8 April 2021) and was conducted in accordance with the Helsinki Declaration of 1975, as revised in 2013.

### 2.1. CBCT Measurements

**Figure 1:**
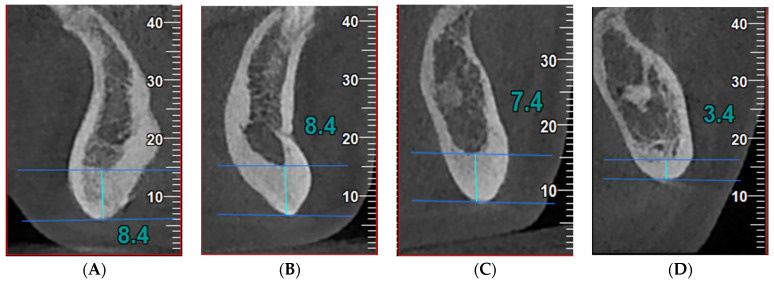
Index measurements in cross-sectional images in a patient with acromegaly: (**A**) (S index, symphysis)—the thickness of the mandibular inferior cortex equidistant from the centers of the right and left mental foramina; (**B**) (A index, anterior)—the thickness of the mandibular inferior cortex 10 mm anterior to the mental foramina; (**C**) (M index, molar)—the thickness of the mandibular inferior cortex 10 mm posterior to the mental foramina; (**D**) (P index, posterior)—the thickness of the mandibular inferior cortex 25 mm posterior to the mental foramina. The high porosity of the bone can be observed, suggesting an impaired bone micro-architecture.

The CBCT images were obtained using NewTom VGi EVO Cone Beam 3D Imaging (CEFLA s.c.—Via Selice Provinciale 23/a Imola, Italy), at 110 kV, 7.5 mA, 3.5 s, pixel size 0.2 mm. The images were reconstructed using NewTom NNT (ISDP©10003:2020 compliant [16] in accordance with EN ISO/IEC 17065:2012 certificate number 2019003109-2 [17]) with Viewer software version 16.0 (see Figure 1).

We analyzed cross-sectional CBCT images in 4 sites identified according to the mental foramen (the standard region for BMD evaluation), as follows:

- Anterior index (A)—the thickness of the inferior mandibular cortex 10 mm anterior from the MF;
- Molar index (M)—the thickness of the inferior mandibular cortex 10 mm posterior from the MF;
- Posterior index (P)—the thickness of the inferior mandibular cortex 25 mm posterior from the MF;
- Symphysis index (S)—the thickness of the inferior mandibular cortex equidistant from the centers of the right and left MF.

### 2.2. Bone Mineral Density Measurements

The sites for the BMD (expressed in grams per square centimeters (g/cm^2^)) measurements were the lumbar spine (LS), the femoral neck (FN), and the total hip (TH). These were performed using DXA (General Electric Prodigy Lunar, Bedford, UK) with enCore Software 10,50,086. The T score (expressed in standard deviations (SDs)) was obtained by comparing the BMD with the peak bone mass of a young adult [15]. All the measurements were performed according to the International Society for Clinical Densitometry (ISCD) [18]. The TBS values in the L1–L4 vertebrae were obtained on DXA images using iNsight Software (v. 2.2.0.0, Medimaps Group SA Headquarters, Geneva, Switzerland).

All the patients were scanned by two different operators but on the same DXA machine.

According to the 2020 AACE (American Association of Clinical Endocrinologists) guidelines, the diagnosis of osteoporosis in postmenopausal women is based on the following criteria [19]:

- T score −2.5 SD or below in the lumbar spine, femoral neck, total proximal femur, or 1/3 radius
- Low-trauma spine or hip fracture (regardless of bone mineral density)
- T score between −1.0 and −2.5 SD and a fragility fracture of the proximal humerus, pelvis, or distal forearm
- T score between −1.0 and −2.5 SD and high FRAX^®^ (fracture risk assessment tool) fracture probability based on country-specific thresholds

### 2.3. Statistical Analysis

We statistically analyzed the patients based on the value of the BMD (lumber spine, femoral neck, total hip), the T score (lumber spine, femoral neck, total hip), and the TBS as continuous values, regardless of the osteoporosis diagnosis at the time of the CBCT evaluation.

We also used binary logistic analysis to divide the patients based on the osteoporosis diagnosis (according to AACE/ACE criteria) [20]. We also employed parametric tests, regression analysis, the *t*-test, Pearson’s correlation coefficient, and Spearman’s rho using IBM SPSS Statistics, version 25 (SPSS Inc., Chicago, IL, USA) for Mac OS.

## 3. Results

The study focused on the relationship between the computed tomography radiomorphometric mandibular indices obtained from cone beam CT (CBCT) and the BMD measurements from the DXA and the TBS assessment in patients with endocrine pathologies that interfere with bone remodeling. The patients’ characteristics, divided by a T score diagnostic of osteoporosis based on the World Health Organization (WHO) criteria (lumbar, femoral and total hip score, less or equal to −2.5 SD) [21], are listed in Table 2 below.

Table 3 shows the mean values of the studied CBCT parameters in the studied patients with secondary causes of low bone mass. It can be observed that the patients with osteoporosis had lower CBCT parameters compared to those with a T score > −2.5. The patients with normal bone micro-architecture (evaluated using the TBS) had higher values of the CBCT indices compared to those with impaired bone micro-architecture.

In this study, both Pearson’s and Spearman correlation coefficients were used to analyze the relationship between the mandibular CBCT indices and the BMD and TBS measurements (Table 4). No significant correlation was found between the DXA and TBS parameters and the symphysis index (S) (*p* > 0.05), indicating that it does not linearly relate to bone density or micro-architecture. The lumbar T score positively correlated (moderate strength) with the anterior (A) and molar (M) indices (r = 0.387 for A index and r = 0.429 for M index, respectively). Almost all the DXA parameters evaluated (T scores and BMD) and the TBS score correlated with the A, M, and P indices. The highest correlation coefficient in the secondary causes group was observed between the A index and the lumbar BMD (r = 0.375, *p* = 0.001) and the P index and the femoral neck BMD (r = 0.38, *p* = 0.001).

Logistic regression analysis was used to evaluate the predictive value of CBCT-derived mandibular in assessing osteoporosis as evaluated by DXA T scores and bone micro-architecture with respect to the TBS values. No significant correlation was observed regarding the quantity mass assessed by DXA-derived parameters. An interesting observation was the predictive value of the A, M, and P indices of the bone micro-architecture evaluated using the TBS, which was statistically significant. The symphysis index does not significantly predict osteoporosis or altered bone micro-architecture.

Table 5 shows the predictions between the causes of secondary osteoporosis. Hypercortisolism seems to be the most predictable cause of secondary osteoporosis using the A, M, and P indices, with the M index being statistically significantly associated with other causes like acromegaly and treatment with aromatase inhibitors.

## 4. Discussion

Osteoporosis is a global public health problem, with fractures being associated with significant morbidity and mortality. Up to 30% of postmenopausal women, >50% of premenopausal women, and between 50% and 80% of men have secondary osteoporosis [1].

Secondary causes of osteoporosis stemming from endocrine disorders intricately affect bone micro-architecture through various pathophysiological mechanisms, often leading to increased fragility and fracture risk, irrespective of BMD [22]; in most of the pathologies, the relationship between the BMD and fracture risk is very different compared to postmenopausal osteoporosis [1,23,24]. For example, primary hyperparathyroidism determines cortical bone thinning and trabecular bone loss, increasing the fracture risk [25,26]. Unlike postmenopausal osteoporosis, which primarily involves a decrease in bone formation due to estrogenic deficiency [3], it causes more severe structural deterioration with an increased fracture risk. On the other hand, in hyperthyroidism, there is an accelerated bone turnover, with the resorptive activity surpassing bone formation and leading to bone loss [2,27]. This results in trabecular thinning and cortical porosity, an impaired bone micro-architecture, which is more dramatic than the gradual decline seen in postmenopausal osteoporosis [1,27].

In our paper, the patients showed significantly lower T scores and BMD values in the osteoporosis group compared to the normal/osteopenia group. The lumbar spine region appears to be particularly affected, with the lowest T scores observed here. This could be due to the rich trabecular bone content in this region, which is more susceptible to metabolic changes caused by the diseases of the included patients [1,22]. Compared to other studies in the literature, the present study evaluated patients with osteoporosis and also osteopenia rather than just comparing osteoporosis versus normal bone mass [15].

Bone densitometry, using dual-energy X-ray absorptiometry, may underestimate the fracture risk in some chronic diseases (like glucocorticoid-induced osteoporosis, type 2 diabetes, and obesity) [22,24,28] and can overestimate the fracture risk in others [1,29]. When evaluating the osseous changes of the jaws of patients with chronic renal failure [30], another cause of secondary low bone mass, it was found that CBCT is a valuable diagnostic tool for the evaluation of osseous findings, the pulp chamber, soft-tissue calcifications, and the mandibular cortical index. This way, it allows the measurement of indices in three dimensions without any superposition.

While DXA remains the gold standard for diagnosing osteoporosis [19,20], the present study highlights the complementary role of CBCT in providing additional insights. The most commonly used quantitative indices for determining low BMD in the MF region, according to the literature, are the panoramic mandibular index (PMI) [31] and the mental index (MI), also known as the mandibular cortical width (MCW) [32]. The new CBCT indices proposed by Barra et al. [15] are similar to the MCW used in panoramic radiographs but are located in different regions of the mandible. As with radiomorphometric indices in panoramic radiographs, these CBCT indices showed lower values in patients with low BMD compared to healthy individuals [33]. In our study, we assessed these indices (10 mm anterior, 10 mm posterior, and 25 mm posterior from the mandibular foramen (MF)) to validate their reliability in the low bone mass of secondary endocrine causes.

This study found significant correlations between the studied CBCT-derived mandibular indices and BMD measurements from DXA and bone micro-architecture assessed using the TBS. Specifically, the A, M, and P indices correlated with the BMD and the TBS in all the included endocrine causes of low bone mass. However, the weaker correlations observed for the T score (lumbar T score and anterior (A) and molar (M) indices: a correlation coefficient of 0.361, for A index, and a correlation coefficient of 0.313 for M index, respectively) indicate the need for further validation in these pathologies.

Across the group included in the study, the CBCT-derived indices were lower in osteoporotic patients compared to those in patients with a normal BMD or osteopenia. This consistent pattern could potentially underscore the role of the studied indices in reflecting variations in the bone micro-architecture and density that are characteristic of osteoporosis. In a study that evaluated the same CBCT indices for assessing the BMD status in 48 postmenopausal women, the M and P indices were significantly lower in osteoporosis than in normal patients (*p* = 0.001 and 0.008, respectively).

Patients with a lumbar T score non-diagnostic for osteoporosis based on the WHO criteria [20] (T score > −2.5 SD) had significantly higher mean CBCT values across all indices compared to those with a lumbar T score ≤ −2.5 SD. Similar trends were observed for the femoral T scores. The patients with a normal or mildly reduced BMD (T score > −2.5 SD) had thicker mandibular cortices in the CBCT-derived measurements compared to those with osteoporosis (T score ≤ −2.5 SD). These results suggest that CBCT can distinguish between different degrees of bone density. In addition to cancellous bone density, the cortical bone thickness at the edentulous site is another factor influencing the initial stability of a future implant [34,35]. When using CBCT to evaluate jawbone quality and measure implant stability after dental implants, Song et al. [36] found that thicker cortical bone layers resulted in greater dental implant stability.

Regarding the prediction value of the CBCT indices for osteoporosis, the anterior and molar indices were not predictors for osteoporosis of secondary causes with regard to the quantity mass assessed by DXA-derived parameters. A very interesting observation was the predictive value of the A, M, and P indices of the bone micro-architecture evaluated using the TBS, which was statistically significant. This was in line with the pathophysiology of secondary causes that affect bone health by interfering with the bone micro-architecture and not the quantity [1,23,24]. This could be important for patients with diabetes mellitus and type 2 diabetes mellitus (T2DM) which are characterized by a normal or high BMD but with an increased risk of fragility fractures [37], so there are other factors that influence this risk [38,39]. Lower TBS values are associated with a higher risk of fragility fractures (independent of BMD), and the altered bone micro-architecture is a significant adjuvant to fracture risk in T2DM [37,38,39].

Hypercortisolism seems to be the most predictable cause of secondary osteoporosis using the A, M, and P indices. The M index is also statistically significantly associated with other causes like acromegaly, taking into consideration that these pathologies, especially Cushing’s, are known to cause devastating effects on bone health. Prolonged exposure to high cortisol levels suppresses osteoblast function and promotes osteoclast activity [40], with the extensive bone micro-architecture deterioration in Cushing’s syndrome often resulting in a higher fracture risk compared to the bone density-related fractures in postmenopausal osteoporosis [41], which are not always directly associated with BMD [2,24].

In the clinical setting, CBCT-derived indices, especially the A, M, and P indices, offer valuable information that can complement traditional DXA measurements. In the cases of patients with secondary endocrine causes of osteoporosis, the utility of CBCT indices, although present, appears to be less pronounced than in postmenopausal women with osteoporosis [15]. This may be due to the heterogeneous nature of the secondary osteoporosis group, which can arise from various underlying conditions affecting bone metabolism. For example, acromegaly, which is characterized by excessive growth hormone (GH) and IGF-1, leads to abnormal bone remodeling and disproportionate bone growth, with thickened cortical bone and thin trabeculae, which paradoxically weakens the overall bone strength. Very interestingly, acromegaly is associated with an abnormal bone structure that increases the risk of fractures independent of the BMD, which is in contrast to the more uniform bone density loss seen in postmenopausal osteoporosis [2].

The strength of our study resides in the important number of patients with secondary endocrine causes of low bone mass and the evaluation of the BMD and not only the T score [42], and in all validated DXA sites, as it can provide more information and a better understanding of the results [22]. The evaluation of the bone micro-architecture using the TBS provides valuable information regarding the bone micro-architecture in addition to the bone quantity, an important tool in assessing bone health, especially in secondary causes of osteoporosis. Specifically, the molar and anterior indices showed robust correlations with the TBS, suggesting that measurements of cortical thickness at these mandibular regions are reliable indicators of trabecular bone micro-architecture. A higher anterior index corresponded to a higher TBS, indicating a superior trabecular structure and a potentially lower fracture risk.

This is the first study to evaluate the possible correlation of CBCT-derived indices and bone quantity and, especially, micro-architecture in patients with endocrine pathologies known to interfere with bone health by affecting the bone remodeling process in different ways.

The limitations of the study include the associated diseases of the patients, which can also interfere with bone mass, like chronic kidney disease, obesity, age at menopause, family history, or different lifestyle habits (tobacco or alcohol use, diet, and physical activity). Additional study limitations are its cross-sectional type and, when dividing the secondary causes group by specific pathology, the relatively small number of patients. This can be explained by the fact that some endocrine diseases that affect bone mass are rare diseases, like endogenous Cushing’s or acromegaly. Future research could expand on these findings by including larger and more diverse populations and exploring longitudinal changes in bone density. The symphysis index showed no significant correlations, suggesting that not all CBCT indices are equally useful. Investigating the cost-effectiveness and accessibility of CBCT in various clinical settings (in Romania, the cost and availability are acceptable considering that it is part of the dental implant protocol) is important when regarding this investigation as a tool for identifying low bone mass. This contributes to the personalized treatment of low bone mass and its cause, resulting in a more stable implant site and a better prognosis for the dental implant.

## 5. Conclusions

In conclusion, our findings underscore the utility of CBCT mandibular indices in the anterior and posterior regions adjacent to the mandibular foramen (MF) for identifying low bone mass in osteoporotic patients. Specifically, the anterior, molar, and posterior indices correlated with the BMD and the TBS in all the included endocrine causes of low bone mass, while the molar and anterior indices showed robust correlations with the TBS. This is valuable information for radiologists and dental practitioners interpreting CBCT images to consider these indices useful for low bone mass. This could be a helpful factor in implant success and peri-implant bone stability after specific treatment of low bone mass and its subjacent endocrine cause.

## Figures and Tables

**Table 1:** Endocrine diseases or metabolic causes of low bone mass.

Primary hyperparathyroidism.
Cushing’s syndrome
Hyperthyroidism
Hypogonadism/premature ovarian insufficiency
Hyperprolactinemia
Acromegaly
Growth hormone deficiency
Diabetes mellitus
Metabolic surgery
Hypophosphatasia
Glucocorticoid-induced
Pregnancy
Genetic diseases

**Table 2:** Patients’ characteristics, postmenopausal women, and endocrine secondary causes based on lumbar, femoral, and total hip T score.

Parameter	T Score ≤ −2.5 *	T Score > −2.5 *
Secondary endocrine causes		
Number	18	65
Age at menopause (years)	48.5 ± 5.57	47.2 ± 5.62
Femoral neck T score (SD)	−2.02 ± 0.74	−0.79 ± 1.25
Total hip T score (SD)	−1.46 ± 0.95	−0.16 ± 0.9
Lumbar T score (L1–L4) (SD)	−2.92 ± 0.36	−0.65 ± 1.02
Femoral neck BMD (g/cm^2^)	0.736 ± 0.09	0.912 ± 0.13
Total hip BMD (L1–L4) (g/cm^2^)	0.827 ± 0.12	0.972 ± 0.27
Lumbar BMD (g/cm^2^)	0.814 ± 0.08	1.108 ± 0.16
TBS score (g/cm^2^)	1.236 ± 0.09	1.313 ± 0.131

* Values are expressed as mean ± standard deviation (SD), NOT based on osteoporosis American Association of Clinical Endocrinology (AACE) criteria. BMD, bone mass density; TBS, trabecular bone score.

**Table 3:** Mean values of the computed tomography parameters on cone beam computed tomography (CBCT) images.

CBCT Parameter	Osteoporosis Based on AACE Criteria	Lumbar T Score > −2.5 SD	Lumbar T Score ≤ −2.5 SD	Low TBS Score (TBS ≤ 1.23)	Intermediate TBS Score (TBS > 1.23 and <1.31)	Normal TBS Score (TBS ≥ 1.31)	Femoral Neck T Score > −2.5 DS	Femoral Neck T Score ≤ −2.5 DS
Secondary endocrine causes								
S	1.95 ± 0.54	2.35 ± 1.35	1.96 ± 0.65	2.01 ± 0.72	2.06 ± 0.7	2.6 ± 1.72	2.34 ± 1.33	2.03 ± 0.77
A	2.45 ± 0.66	2.75 ± 1.09	2.44 ±0.62	2.51 ± 0.76	2.52 ± 0.82	2.95 ± 1.3	2.71 ± 1.08	2.53 ± 0.74
M	2.36 ± 0.72	2.58 ± 0.8	2.17 ± 0.67	2.34 ± 0.8	2.15 ± 0.76	2.87 ± 0.98	2.56 ± 0.93	2.35 ± 1
P	2.36 ± 0.63	2.6 ± 0.8	2.37 ± 0.62	2.39 ± 0.77	2.3 ± 0.71	2.79 ± 0.79	2.59 ± 0.79	2.16 ± 0.79

SD, standard deviation; CBCT, cone beam computed tomography; TBS, trabecular bone score, anterior (A)—10 mm anterior from the mental foramina (MF); molar (M)—10 mm posterior from the MF; posterior (P)—25 mm posterior from the MF; symphysis (S) equidistant from the centers of the right and left MF.

**Table 4:** Correlations between CBCT parameters and bone quantity and micro-architecture parameters in patients with secondary endocrine causes of osteoporosis versus postmenopausal women.

Parameters for Correlations ^†^	*S*	*A*	*M*	*P*
Pearson’s correlation ^†^				
Secondary endocrine causes				
Lumbar T score *	0.01, *p* = 0.931	**0.361, *p* = 0.001**	**0.313, *p* = 0.004**	**0.229, *p* = 0.043**
Femoral neck T score *	0.129, *p* = 0.25	0.183, *p* = 0.10	**0.228, *p* = 0.041**	**0.33, *p* = 0.003**
Total hip T score *	0.10, *p* = 0.33	0.2, *p* = 0.063	**0.232, *p* = 0.037**	**0.275, *p* = 0.014**
TBS score ^#^	0.127, *p* = 0.28	**0.251, *p* = 0.031**	**0.316, *p* = 0.006**	0.182, *p* = 0.12
Lumbar BMD **	0.021, *p* = 0.85	**0.375, *p* = 0.001**	**0.319, *p* = 0.004**	**0.232, *p* = 0.04**
Femoral neck BMD **	0.191, *p* = 0.088	**0.219, *p* = 0.049**	**0.289, *p* = 0.009**	**0.38, *p* = 0.001**
Total hip BMD **	0.166, *p* = 0.138	**0.251, *p* = 0.024**	**0.325, *p* = 0.003**	**0.322. *p* = 0.004**
Spearman’s rho ^†^				
Secondary causes				
Lumbar T score *	0.193, *p* = 0.084	0.206, *p* = 0.066	**0.252, *p* = 0.023**	**0.273, *p* = 0.015**
Femoral neck T score *	0.173, *p* = 0.122	**0.23, *p* = 0.039**	**0.252, *p* = 0.023**	**0.350, *p* = 0.002**
Total hip T score *	**0.231, *p* = 0.038**	**0.241, *p* = 0.03**	**0.237, *p* = 0.033**	**0.252, *p* = 0.025**
TBS score ^#^	0.22, *p* = 0.06	**0.212, *p* = 0.07**	**0.290, *p* = 0.012**	0.2, *p* = 0.088
Lumbar BMD **	0.197, *p* = 0.078	**0.21, *p* = 0.06**	**0.261, *p* = 0.019**	**0.277, *p* = 0.013**
Femoral neck BMD **	**0.247, *p* = 0.026**	**0.270, *p* = 0.015**	**0.298, *p* = 0.007**	**0.419, *p* < 0.0001**
Total hip BMD **	**0.27, *p* = 0.015**	**0.262, *p* = 0.018**	**0.337, *p* = 0.002**	**0.299, *p* = 0.008**

^†^ Significant at the 0.05 level *t*-test (two-tailed), * expressed as standard deviations, ** expressed as g/cm^2^, ^#^ expressed as continuous values. BMD, bone mass density; TBS, trabecular bone score, expressed as g/cm^2^; anterior (A)—10 mm anterior from the mental foramina (MF); molar (M)—10 mm posterior from the MF; posterior (P)—25 mm posterior from the MF; symphysis (S) equidistant from the centers of the right and left MF.

**Table 5:** Predictions between causes of secondary type osteoporosis.

Parameters	Variable	Odds Ratio	Model’s Sig.
Diabetes mellitus (*n* = 14) in patients with osteoporosis	S	1.03, 95% CI (0.937, 1.131)	*p* = 0.54
	A	0.989, 95% CI (0.909, 1.076)	*p* = 0.79
	M	1.014, 95% CI (0.938, 1.096)	*p* = 0.72
	P	1.018, 95% CI (0.937, 1.106)	*p* = 0.67
Cushing’s (*n* = 8) in patients with osteoporosis	S	1.080, 95% CI (0.96, 1.215)	*p* = 0.2
	A	**1.120, 95% CI (0.99, 1.268)**	***p* = 0.053**
	M	**1.125, 95% CI (1.011, 1.251)**	***p* = 0.023**
	P	**1.162, 95% CI (1.030, 1.312)**	***p* = 0.009**
Acromegaly (*n* = 4) in patients with osteoporosis	S	1.047, 95% CI (0.890, 1.231)	*p* = 0.58
	A	0.952, 95% CI (0.816, 1.111)	*p* = 0.52
	M	**1.179, 95% CI (1.011, 1.375)**	***p* = 0.023**
	P	1.046, 95% CI (0.903, 1.210)	*p* = 0.55
Hyperthyroidism (*n* = 7) in patients with osteoporosis	S	0.940, 95% CI (0.814, 1.086)	*p* = 0.38
	A	0.997, 95% CI (0.754, 1.064)	*p* = 0.16
	M	0.983, 95% CI (0.882, 1.096)	*p* = 0.75
	P	0.938, 95% CI (0.831, 1.059)	*p* = 0.28
Aromatase inhibitors treatment in patients with osteoporosis (*n* = 6)	S	1.028, 95% CI (0.89, 1.178)	*p* = 0.68
	A	1.035, 95% CI (0.913, 1.172)	*p* = 0.58
	M	**1.120, 95% CI (0.995, 1.260)**	***p* = 0.052**
	P	1.10, 95% CI (0.961, 1.261)	*p* = 0.15
Hyperthyroidism (*n* = 4) in patients with osteopenia and normal bone mass	S	0.816, 95% CI (0.614, 1.085)	*p* = 0.078
	A	0.896, 95% CI (0.754, 1.064)	*p* = 0.16
	M	**0.841, 95% CI (0.684, 1.034)**	***p* = 0.05**
	P	0.933, 95% CI (0.8, 1.088)	*p* = 0.36
Diabetes mellitus (*n* = 25) in patients with osteopenia and normal bone mass	S	1.035, 95% CI (0.988, 1.083)	*p* = 0.086
	A	0.987, 95% CI (0.942, 1.034)	*p* = 0.57
	M	0.970, 95% CI (0.918, 1.025)	*p* = 0.261
	P	0.951, 95% CI (0.893, 1.014)	*p* = 0.117
Cushing’s (*n* = 7) in patients with osteopenia and normal bone mass	S	0.944, 95% CI (0.837, 1.065)	*p* = 0.28
	A	0.973, 95% CI (0.890, 1.062)	*p* = 0.51
	M	0.964, 95% CI (0.877, 1.060)	*p* = 0.43
	P	1.008, 95% CI (0.905, 1.122)	*p* = 0.88
Aromatase inhibitors treatment (*n* = 9) in patients with osteopenia and normal bone mass	S	0.969, 95% CI (0.883, 1.063)	*p* = 0.44
	A	0.985, 95% CI (0.916, 1.059)	*p* = 0.66
	M	0.986, 95% CI (0.911, 1.067)	*p* = 0.72
	P	1.041, 95% CI (0.95, 1.14)	*p* = 0.38
Acromegaly (*n* = 8) in patients with osteopenia and normal bone mass	S	**1.083, 95% CI (0.996, 1.177)**	***p* = 0.062**
	A	**1.069, 95% CI (1.006, 1.136)**	***p* = 0.019**
	M	**1.095, 95% CI (1.013, 1.184)**	***p* = 0.017**
	P	1.027, 95% CI (0.934, 1.130)	*p* = 0.58

Anterior (A)—10 mm anterior from the mental foramina (MF); molar (M)—10 mm posterior from the MF; posterior (P)—25 mm posterior from the MF; symphysis (S) equidistant from the centers of the right and left MF.

## Data Availability

The raw data supporting the conclusions of this article will be made available by the authors on request.
